# Submucosal Lipoma in the Rectum Found Incidentally During Colonoscopy Screening: A Case Report

**DOI:** 10.7759/cureus.56587

**Published:** 2024-03-20

**Authors:** Mohammad Kloub, Earyn Calvis, Bavly Abdelmesih, Rachel Milia, Raed Atiyat, Theodore Jr Dacosta

**Affiliations:** 1 Internal Medicine, Saint Michael's Medical Center, Newark, USA; 2 Medicine, Saint George’s University, School of Medicine, New York, USA; 3 College of Osteopathic Medicine, University of New England College of Osteopathic Medicine, Biddeford, USA; 4 Internal Medicine, Division of Gastroenterology and Hepatology, Saint Micheal’s Medical Center, Newark, USA; 5 Gastroenterology and Hepatology, Saint Michael's Medical Center, Newark, USA

**Keywords:** polyp, colonoscopy, tumor, rectum, lipoma

## Abstract

Gastrointestinal (GI) tract lipomas are a benign entity of GI tumors. In this case report, we present a 53-year-old patient who was found to have a rectal lipoma incidentally in an asymptomatic middle-aged female during a routine colonoscopy screening. The tumor was excised during colonoscopy and sent for histopathology, which confirmed the diagnosis. We also provide a literature review of GI lipomas, focusing on their occurrence in the rectum.

## Introduction

Lipomas, characterized by benign soft tissue tumors made up of adipocytes, are non-epithelial, slow-growing tumors that present as non-tender, mobile, rubbery masses. While most lipomas are commonly found on the skin, including the head, neck, shoulders, and back, their presence in the gastrointestinal (GI) tract is exceedingly rare [1]. Within the GI tract, lipomas are typically found within the esophagus, small intestine, and stomach [2]. Small intestine lipomas make up 25 percent of GI lipomas; specifically, the most common site is within the ileum. Lipomas within the colon contribute to 65 to 75 percent of GI lipomas and are usually found incidentally through the use of colonoscopy. Most of these cases are within the cecum and ascending colon [3]. These tumors can locally cause nerve compression and enlarge to compress other surrounding nearby structures. In addition, these lipomas are symptomatic after the GI tract undergoes luminal obstruction, intussusception, rectal prolapse, and if bleeding occurs [2]. In this case report, we describe a rectal submucosal lipoma found incidentally on colonoscopy via polyp removal, and we also provide a comprehensive literature review on lipomas, with a focus on their occurrence in the GI tract.

## Case presentation

A 53-year-old female with a past medical history of prediabetes presented to the clinic for a routine colonoscopy screening for colon cancer. She denied any alarming GI symptoms and denied previous colonoscopies. The patient denied the use of tobacco, alcohol, and illicit drugs. She did not have any active medications. Past surgical history included ovarian cystectomy 30 years prior. Family history was non-contributory. Vitals on presentation revealed a temperature of 98F, a heart rate of 91 beats/min, a respiratory rate of 16 breaths/min, a blood pressure of 134/91 mmHg, and a BMI of 31.17 kg/m^2^. Physical examination was unremarkable. 

**Figure 1:**
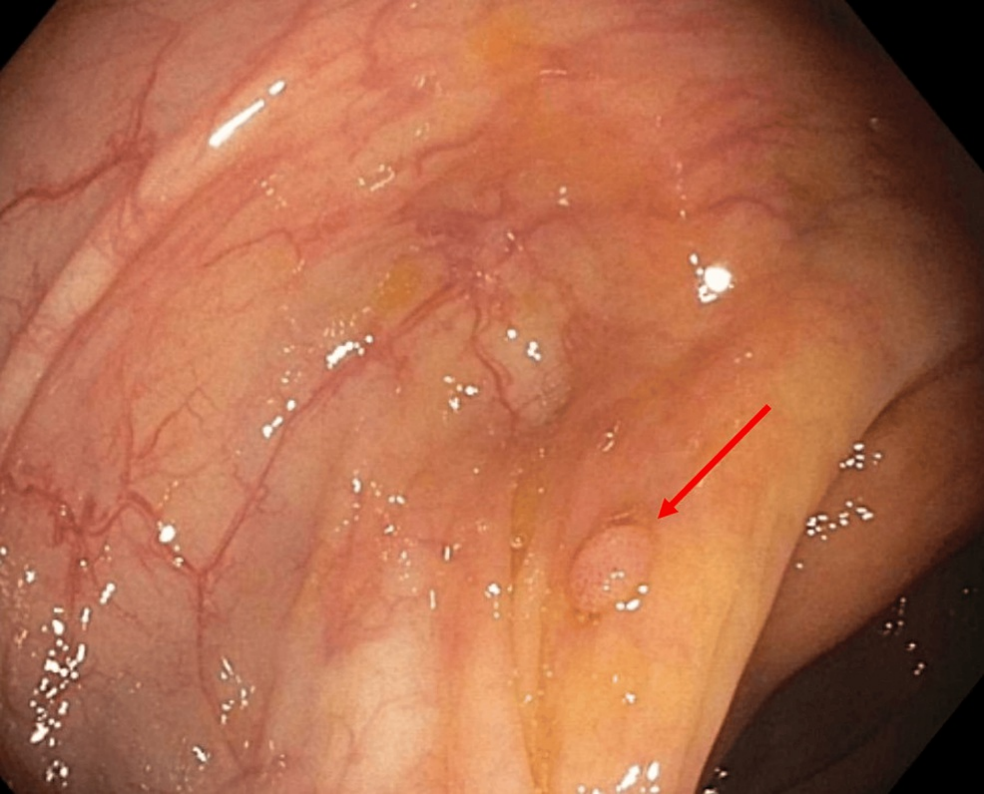
A diminutive sessile polyp in the ascending colon (red arrow).

**Figure 2:**
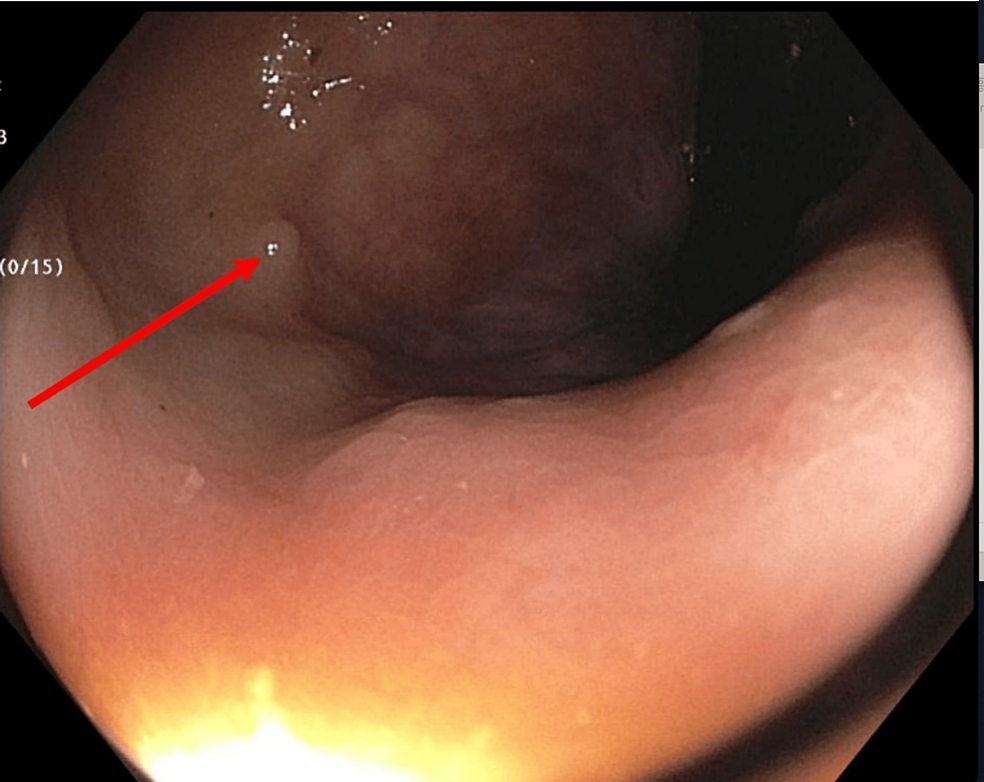
A diminutive sessile polyp in the rectum (red arrow).

**Figure 3:**
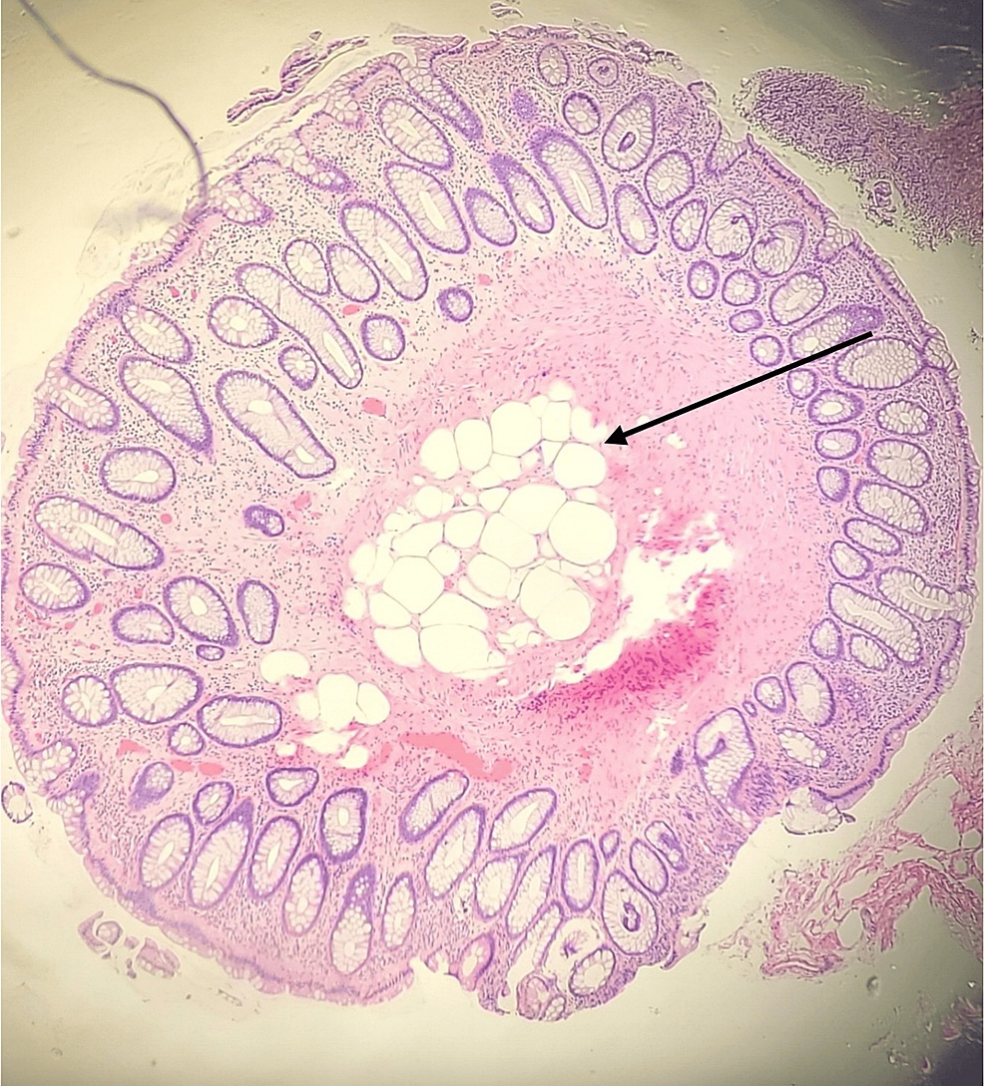
A histopathology of the rectal polyp revealed polypoid mucosa with nodular adipose tissue (black arrow), mainly in the submucosa, with infiltration into the mucosa consistent with submucosal lipoma.

The colonoscopy revealed hemorrhoids, one small-mouthed diverticulum in the cecum, a diminutive sessile polyp in the ascending colon (Figure 1), and a diminutive sessile polyp in the rectum (Figure 2). Both polyps in the ascending colon and rectum were removed with jumbo cold forceps. Resection and retrieval were complete, and there was no estimated blood loss. The patient had an uncomplicated clinical course and was discharged the same day. Pathology of the rectal polyp revealed polypoid mucosa with nodular adipose tissue, mainly in the submucosa, with infiltration into the mucosa consistent with submucosal lipoma (Figure 3).

## Discussion

Lipomas are common benign tumors that are composed of adipose cells coated by a fibrous capsule. They are usually found on the skin, most commonly on the head, neck, back, armpits, and shoulders. However, they can also occur at any site inside of the body, including the GI tract. GI lipomas rise from the submucosa layer and bulge into the lumen [4]. They are usually asymptomatic and are found incidentally during colonoscopy screening or other imaging modalities. However, if enlarged enough (usually larger than 2 cm), they can present with obstruction symptoms, abdominal pain, and ulceration [5]. 

GI lipomas form about 3% of gastrointestinal tumors. The most common sites for GI lipomas are the colon and the small intestines, with a prevalence of 60-75% and 30%, respectively [6]. More specifically, rectal lipomas constitute the smallest percentage of GI lipomas, about 3.40% of all colorectal lipomas, which makes it the most uncommon site for GI lipomas. Although the exact cause and pathophysiology of colorectal lipoma are unknown, some research points to possible connections between the disease and issues with intestinal and systemic fat metabolism as well as malnutrition [7,8].

Under colonoscopy, a lipoma would present as a pink mass with a smooth surface and intact capsule. Some clinical features can suggest a lipoma, which includes a “cushion sign,” “tent effect,” and “naked fat sign”. A cushion sign is having pressure marks on the lesion after pressing on it using forceps. The tent effect is being able to grasp the mucosa away from the mass, causing the internal mass to protrude. The naked fat sign refers to the protruding of the underlying adipose tissue following multiple biopsies removing the overlying mucosa. However, the final definitive diagnosis is confirmed by the histopathology report due to the different morphologies of the various malignancies [8]. 

Management depends on whether the patient is symptomatic or asymptomatic. Small lipomas (<2cm) in asymptomatic patients would be managed with observation and conservative management. However, for symptomatic patients, surgical excision through endoscopic resection or surgical removal would be preferred [4].

## Conclusions

GI lipomas, characterized by benign adipose cell tumors, are most commonly asymptomatic GI tumors. Although they most commonly occur in the colon, they can present in the rectum. This case report underscores the importance of considering GI lipomas, even in unusual locations like the rectum, when dealing with GI polyps. Prompt intervention and follow-up colonoscopy, if indicated, are crucial for the successful management and detection of early recurrence.
